# Microgravity-induced alterations in the molecular and cellular characteristics of brain tumors: a systematic review

**DOI:** 10.1097/MS9.0000000000003453

**Published:** 2025-05-30

**Authors:** Mrinmoy Kundu, Rafael Tiza Fernandes, Anuj Kumar Sharma, Hala Ibrahim Thaalibi, Andrew Awuah Wireko, Toufik Abdul-Rahman

**Affiliations:** a Institute of Medical Sciences and SUM Hospital, Bhubaneswar, India; b Toufik’s World Medical Association, Sumy, Ukraine; c Department of Neurosurgery, ULS, São José, Lisbon, Portugal; d Center for Aerospace Medicine Studies, Faculty of Medicine, University of Lisbon, Lisbon, Portugal; e St. John’s Medical College Hospital, Bangalore, India; f Beirut Arab University Faculty of Medicine, Beirut, Lebanon

**Keywords:** brain tumors, cellular responses, glioblastoma multiforme, microgravity, therapeutic applications

## Abstract

**Background:**

Microgravity profoundly impacts various biological functions, including those crucial to tumorigenesis. Investigating the effects of microgravity on brain tumors is pivotal for understanding tumor biology and developing novel therapeutic strategies. This delineates the molecular and cellular characteristics of brain tumor cells under microgravity conditions.

**Methodology:**

The authors systematically reviewed the literature to identify relevant studies.

**Results:**

Microgravity has been shown to (i) induce morphological changes, where cultured glioma cells showed inhibited invasion through formation of multicellular aggregates, and ANGM5 glioblastoma multiforme (GBM) cell line showed 3D multicellular spheroid formation and loss of adhesion; (ii) inhibit cellular proliferation generally, as well as reduce invasion and migration in U87 GBM cells, however, cell viability remained high in A-172 GBM cells and human umbilical vein endothelial cells; (iii) increase DNA damage, evidenced by increased comet tail length and expression of phosphorylated γ-H2A.X, and activate apoptotic pathways in GBM and microglial cells; (iv) alter signaling pathways and protein expression through activation of ERK1/2 and AKT, altering the expression of GSK3β, Bax, and Bcl-2 in microglial and GBM cells, and through decreasing expression of vinculin and active Yap1 in GBM cells, and that of of Yap1 and ZO-1 in endothelial cells; and (v) increase chemosensitivity of GBM cells to cisplatin.

**Conclusion:**

Studies have shown that, across several rotary cell culture systems (RCCSs), random-position machines (RPMs), and 3D bioprinted GBM on-a-chip models, microgravity has consistently been proven to have profound effects on GBM cells. This further drills the potential role offered by microgravity as a promising anti-cancer agent in brain tumors, especially GBM.

## Introduction

Microgravity, which is nearly the zero gravity that is felt in space or can be replicated through simulation on Earth, has a significant impact on a variety of essential biological functions. These include complex features like changes in cell shape, adjustments to the rates of cellular proliferation, adjustments to migration patterns, and notable effects on gene expression^[1,2]^. The investigation of the effects of microgravity on tumor cells is particularly important and is a focus of scientific research. Investigating these effects could shed new light on the complex processes regulating tumorigenesis and tumor suppression^[3,4]^. Additionally, it offers a path for the discovery of possible therapeutic approaches in the field of cancer therapy. In this regard, brain tumors stand out as a topic of particular interest and concern because of their location, invasiveness, and resistance to traditional therapies. Understanding the cellular and molecular processes driving the growth of brain tumors is essential to creating successful treatment strategies. The ability to study these mechanisms in the context of microgravity presents a special chance to find new targets or checkpoints that might be used therapeutically. In the context of CNS tumors, a wide range of neoplasms arise from various CNS cell types and display a range of features from benign to malignant or borderline. Notably, the prognosis for high-grade glial tumors is typically fatal, except for pilocytic astrocytoma^[5]^. Males are more likely than females to develop malignant brain tumors, whereas adult females are more likely to develop benign meningiomas^[5]^. The annual incidence of primary malignant CNS tumors varies globally, ranging from 2.1 to 5.8 per 100 000 individuals^[5]^. Gaining knowledge about how microgravity affects the molecular and cellular properties of these various kinds of brain tumors may help uncover new treatment options as well as important biological insights. This systematic review aims to comprehensively synthesize the existing knowledge and identify potential avenues for future research in this emerging field.

**HIGHLIGHTS**

- Microgravity profoundly impacts various biological functions, including those crucial to tumorigenesis.
- Microgravity alters cell morphology, suppresses proliferation, induces DNA damage and apoptosis, modulates signaling pathways and protein expression, and enhances chemosensitivity in glioblastoma multiforme cells.
- Future studies should focus on elucidating the molecular mechanisms underlying the cellular responses to microgravity, as well as investigating the impact of microgravity on other aspects of brain tumor biology, such as angiogenesis, immune evasion, and stem cell properties.

## Methodology

### Data sources and search strategy

A comprehensive literature search was conducted to identify relevant studies that examined the molecular and cellular characteristics of brain tumor cells under microgravity conditions, either in actual spaceflight or simulated microgravity platforms. The search was performed in the following electronic databases: PubMed, Scopus, Web of Science, and Cochrane Library. The search strategy included a combination of relevant Medical Subject Headings (MeSH) terms and keywords related to “brain tumors,” “microgravity,” “spaceflight,” and “simulated microgravity.” The specific search queries used in each database were tailored to the respective database’s search syntax and functionality. The search was conducted without any restrictions on publication date or language; however, only studies published in English were considered for inclusion in the review.

### Inclusion and exclusion criteria

Studies were eligible for inclusion if they met the following criteria: (1) the study was a primary research article that examined the molecular and cellular characteristics of brain tumor cells in microgravity, either in actual spaceflight or simulated microgravity platforms; (2) the study used human or animal brain tumor cells, either from cell lines or primary tumors, with the type and grade of the brain tumor cells specified; and (3) the study was published in English in a peer-reviewed journal. Studies were excluded if they did not meet these criteria, specifically if they were not primary research articles using omics approaches, did not use human or animal brain tumor cells, or were not published in English in a peer-reviewed journal.

### Study selection and data extraction

The study selection process followed the Preferred Reporting Items for Systematic Reviews and Meta-Analyses (PRISMA) guidelines. After removing duplicates, the titles and abstracts of the identified records were screened independently by two reviewers (M.K. and R.T.F.) to assess their eligibility for inclusion in the review. Full-text articles of the potentially eligible studies were then retrieved and evaluated against the inclusion and exclusion criteria.

Data extraction was performed following the guidelines and recommendations from the Cochrane Handbook for Systematic Reviews of Interventions and the PRISMA reporting guidelines. A standardized data extraction form was developed and piloted by two reviewers (M.K. and R.T.F.) using a sample of studies, and the form was revised and finalized based on the feedback and results of the pilot. The data extraction form included items related to study characteristics, brain tumor characteristics, and microgravity characteristics. Two reviewers (M.K. and R.T.F.) independently extracted the data from the eligible studies using the data extraction form.

### Data analysis and reporting

The included studies were assessed for methodological heterogeneity, and it was determined that they were too diverse in their methods to combine them in a meta-analysis. Therefore, a qualitative summary of the findings was performed instead, following the SWiM (synthesis without meta-analysis) guidelines for SWiM. The qualitative synthesis aimed to provide a comprehensive overview of the molecular and cellular alterations observed in brain tumor cells exposed to microgravity conditions, as reported by the included studies. The findings were organized and presented based on the various aspects of brain tumor biology such as gene expression profiles, protein abundance and post-translational modifications, metabolic pathways, and cellular processes (e.g., proliferation, apoptosis, migration, and invasion).

## Result

### Eligible studies/study selection

**Figure 1.**
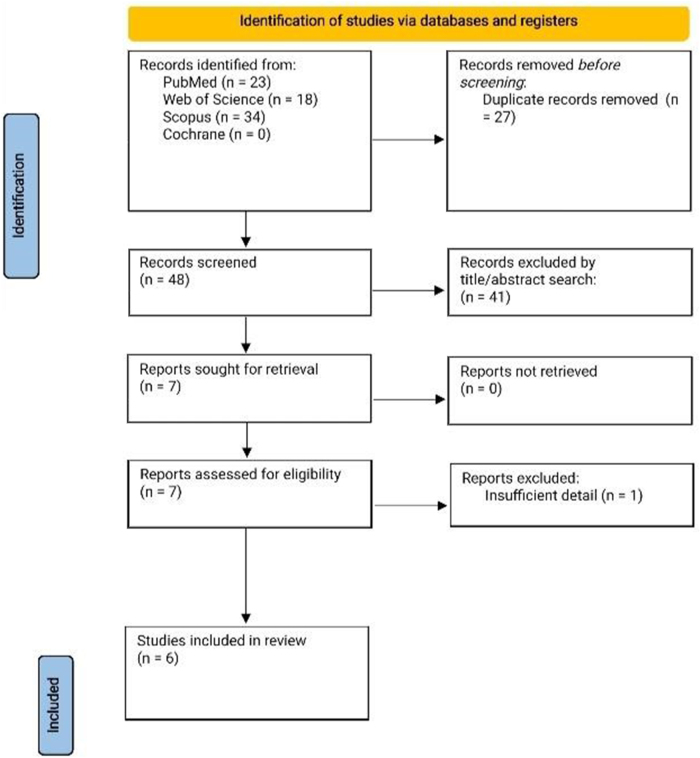
PRISMA flow chart used for the selection of studies.

The systematic literature search yielded a total of 75 records from various databases (Fig. 1), including PubMed (*n* = 23), Scopus (*n* = 34), Web of Science (*n* = 18), and Cochrane (*n* = 0). After removing duplicates (*n* = 27), 48 records remained for screening. Following the title and abstract screening, 41 records were excluded, leaving 7 full-text articles for eligibility assessment. One article was further excluded due to insufficient detail, resulting in a final inclusion of 6 studies for the systematic review (Fig. 1).

### Study characteristics

**Figure 2.**
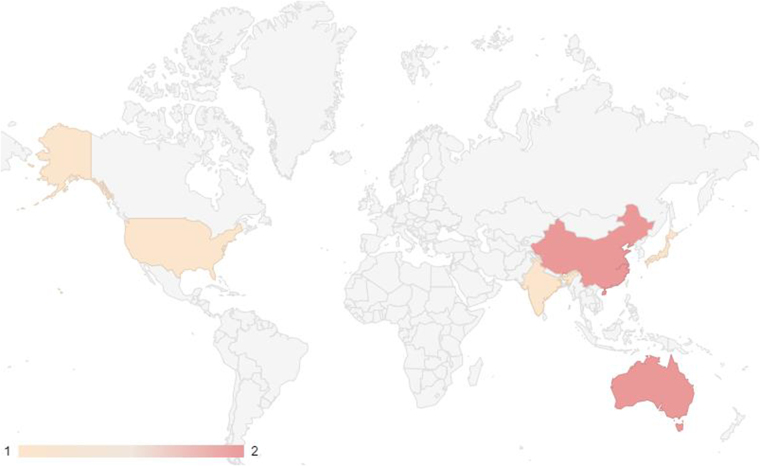
Geographical distribution of the included studies.

**Figure 3.**
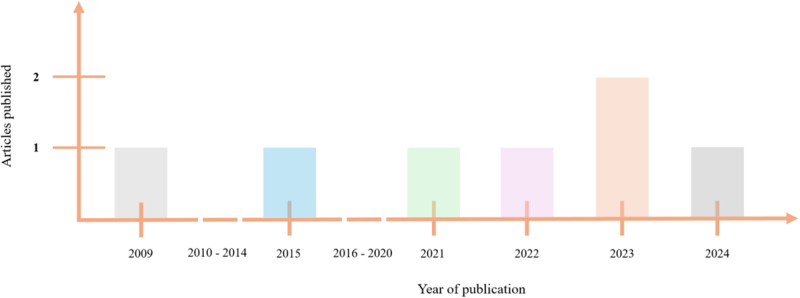
Publication trend of the included studies.

The systematic review included a total of seven studies investigating the effects of microgravity conditions on various brain tumor cells. These studies originated from various countries across the globe (Fig. 2). The majority of the studies were conducted in the United States (*n* = 2) and Australia (*n* = 2), while China, India, and Japan each contributed one study. Regarding the publication timeline, the included studies spanned from 2009 to 2024, reflecting the growing interest and research efforts in this field over the past decade and a half (Fig. 3). Notably, there has been an increase in the number of studies published in recent years, with four studies published in 2022, 2023, and 2024, suggesting an escalating momentum in exploring the interplay between microgravity and brain tumor biology.

The studies originated from diverse geographical locations (Fig. 2), indicating a global recognition of the importance of this research area. Additionally, the increasing trend in the number of publications over the past few years (Fig. 3), highlights the burgeoning nature of this field and the potential for further advancements in our understanding of microgravity’s impact on brain tumor cells.

### Cell morphology and aggregation

Several studies reported morphological changes and the formation of multicellular spheroids or aggregates in glioblastoma multiforme (GBM) cells under simulated microgravity conditions. Glioma cells cultured in a 3D-printed vascularized GBM-on-a-chip model exhibited inhibited invasion and aggregation into the surrounding microenvironment when exposed to microgravity^[6]^. Similarly, the ANGM5 GBM cell line showed a loss of adhesion and the formation of 3D multicellular spheroids after 24 hours of simulated zero gravity^[7]^.

### Cell proliferation and viability

Microgravity conditions generally suppressed cell proliferation in GBM cells. Shi *et al* observed reduced invasion and migration potentials in U87 GBM cells under modeled microgravity^[8]^. Takeda *et al* reported a significant reduction in cell proliferation and decreased mitochondrial activity in various GBM cell lines (D54MG, U251MG, and T98G) exposed to simulated microgravity^[9]^. However, Silvani *et al* found that while cell proliferation was significantly reduced, cell viability remained high in A-172 GBM cells and human umbilical vein endothelial cells cultured in a microgravity-on-a-chip platform^[10]^.

### DNA damage and apoptosis

Exposure to simulated microgravity induced DNA damage and activated apoptotic pathways in GBM cells. Singh *et al* observed increased DNA damage, indicated by increased comet tail length and expression of phosphorylated γ-H2A.X, as well as differential activation of DNA damage response signaling pathways in microglial and GBM cells under simulated microgravity^[11]^. Additionally, enhanced apoptosis was evident through increased DNA fragmentation, altered expression of pro-apoptotic and anti-apoptotic proteins, and activation of caspase-3 and poly (ADP-ribose) polymerase cleavage.

### Signaling pathways and protein expression

Microgravity conditions influenced various signaling pathways and protein expression in GBM cells. Singh et al. reported the activation of ERK1/2 and AKT signaling pathways, as well as altered expression of GSK3β, Bax, and Bcl-2 in microglial and GBM cells under simulated microgravity^[11]^. Silvani et al. observed decreased expression of vinculin and active Yap1 in GBM cells, and compromised expression of Yap1 and ZO-1 in endothelial cells when exposed to microgravity conditions in their GBM-on-a-chip model^[6]^.

### Chemosensitivity

Takeda *et al* found that microgravity-exposed GBM cells exhibited increased sensitivity to the chemotherapeutic agent cisplatin (CDDP) compared to control cells under normal gravity conditions^[9]^.

In summary, the systematic review revealed that microgravity conditions induced significant alterations in the molecular and cellular characteristics of brain tumor cells, particularly GBM. These changes include altered cell morphology, suppressed proliferation, increased DNA damage and apoptosis, modulation of signaling pathways and protein expression, and increased chemosensitivity. These findings highlight the potential impact of microgravity environments on brain tumor biology and underscore the importance of further investigating these effects for therapeutic applications.

## Discussion

The findings of this systematic review provide insight into how microgravity affects the molecular and cellular characteristics of brain tumors, particularly glioblastoma multiforme. Several key observations and implications emerge as a result of combining evidence from multiple studies. One of the most striking findings is the morphological changes observed in GBM cells in microgravity. The formation of multicellular spheroids or aggregates, as reported in studies by Silvani *et al* ^[6]^ and Altaie *et al* ^[7]^, is consistent with observations in other cancer cell types, such as thyroid cancer cells^[12]^ and breast cancer cells^[13]^. This phenomenon is thought to be a cellular response to altered environmental conditions that mimics the tumor microenvironment and promotes cell–cell interactions^[14]^. However, Silvani *et al* observed inhibited invasion and aggregation of GBM cells in their 3D-printed vascularized GBM-on-a-chip model, implying that the effects of microgravity on cell behavior may vary depending on the context^[6]^. Shi *et al*^[8]^, Takeda *et al*^[9]^, and Silvani *et al* ^[10]^ found that microgravity suppresses cell proliferation, which is consistent with findings in other cell types^[15-17]^. This effect has been linked to disruptions in cellular signaling pathways, cytoskeletal organization, and nutrient transport mechanisms in the absence of gravity^[18]^. Interestingly, while cell proliferation was reduced, Silvani *et al*found that cell viability remained high in their microgravity-on-a-chip model, implying that the effects of microgravity on cell survival and proliferation may differ^[10]^. Singh *et al*^[11]^ found that simulated microgravity conditions induce DNA damage and activate apoptotic pathways in GBM cells, which is consistent with findings in other cancer cell types^[19,20]^. The differential activation of DNA damage response signaling pathways in microglial and GBM cells demonstrates cell-type-specific responses to microgravity. Furthermore, the involvement of apoptotic pathways, such as altered expression of pro- and anti-apoptotic proteins and caspase activation, suggests that microgravity may make GBM cells more susceptible to apoptosis^[19]^. Singh *et al*^[11]^ found that microgravity conditions affect signaling pathways like ERK1/2, AKT, and GSK3β, which is consistent with observations in other various cell types exposed to microgravity conditions^[21,22]^. These pathways are known to play crucial roles in cell survival, proliferation, and migration, and their dysregulation under microgravity conditions may contribute to the observed cellular effects. Additionally, the altered expression of cytoskeletal and cell adhesion proteins, such as vinculin, Yap1, and ZO-1, as reported by Silvani *et al*, further supports the notion that microgravity disrupts cytoskeletal organization and cell–cell interactions, which may impact tumor progression and metastasis^[6]^. Interestingly, Takeda *et al*reported increased sensitivity of microgravity-exposed GBM cells to the CDDP. This observation aligns with findings in other cancer cell types, such as leukemia^[23]^ and gastric cancer cells^[24]^, suggesting that microgravity conditions may enhance the efficacy of certain chemotherapeutic agents. This phenomenon has been attributed to various mechanisms, including alterations in drug uptake, cellular metabolism, and the induction of oxidative stress^[25]^. While the studies included in this systematic review provide valuable insights, it is important to acknowledge some limitations. First, the majority of the studies employed simulated microgravity conditions using various devices, such as random-positioning machines (RPMs), clinostats, and rotary cell culture systems (RCCS). These simulations may not fully recapitulate the complex environment of actual microgravity experienced during spaceflight. Additionally, the duration of microgravity exposure varied among studies, ranging from a few hours to several days, which may influence the observed cellular responses. Furthermore, the studies utilized different GBM cell lines, which may exhibit varying responses to microgravity conditions due to their inherent genetic and phenotypic heterogeneity. Despite these limitations, the findings of this systematic review provide a foundation for further exploration of the potential therapeutic applications of microgravity conditions in brain tumor management. The observed effects on cell morphology, proliferation, DNA damage, apoptosis, and chemosensitivity suggest that microgravity environments may offer novel strategies for modulating tumor progression and enhancing the efficacy of existing treatments.

## Conclusion

When simulated in terrestrial settings, microgravity has been shown to consistently alter cells in CNS tumors, especially glioblastoma multiforme, through disrupting cytoskeletal architecture, inhibiting proliferation, inducing DNA damage and cellular apoptosis, rewiring survival pathways (ERK/AKT, Hippo, and GSK3β), and increasing chemosensitivity to cisplatin. Studied through several platforms such as RPMs, rotary bioreactors, and 3D GBM-on-a-chip models, microgravity has been proven to be an effective modulator of different pathogenic mechanisms that drive malignant cells. Some limitations exist; the aforementioned devices may not have simulated the precise microgravity experienced during spaceflight. Furthermore, neither of the studies applied microgravity for equal and consistent durations and on the same cell lines. Regardless, the evidence points toward microgravity being a promising and effective agent in CNS tumor management, opening the door for future research to further and accurately validate these findings clinically.

## Data Availability

No new data was generated.
